# Patterns of care contacts in the final year of life among opioid overdose fatalities in southern Sweden: a latent class analysis

**DOI:** 10.1186/s12954-024-01101-y

**Published:** 2024-10-18

**Authors:** Björn Johnson, Lisa Andersson, Helene Jacobsson, Ardavan M. Khoshnood

**Affiliations:** 1 School of Social Work, Lund University, Lund, Sweden; 2 Department of Social Work, Malmö University, Malmö, Sweden; 3 Clinical Studies Sweden – Forum South, Skåne University Hospital, Malmö, Sweden; 4 Emergency Medicine, Department of Clinical Sciences Malmö, Lund University, Skane University Hospital, Malmö, Sweden

**Keywords:** Opioid overdose fatalities, Opioid use disorder, Opioid agonist treatment, Substance use treatment, Overdose prevention

## Abstract

**Background:**

Understanding the heterogeneity of opioid overdose fatalities is critical to developing effective preventive interventions. This study examines patterns of care contacts among people who subsequently died from opioid overdose. The aim was to identify distinct groups of deceased individuals, based on their contacts with different care agencies in their last year of life.

**Methods:**

A retrospective registry study was conducted in Skåne, Southern Sweden. All recorded opioid overdose fatalities during the study period were included, *n* = 191. Latent class analysis was used to identify patterns of care contacts in the last year of life.

**Results:**

Three distinct classes were identified: “Few care contacts,” with limited interaction with any services; “Social service contacts,” comprising individuals who predominantly had contacts with the social services and, to a lesser extent, with prison and probation services; and “Numerous care contacts,” with extensive contacts with both healthcare and social services. The “few care contacts” class comprises about half of the population. This is an important finding, since this group has not been clearly visible in previous research. The analysis indicates significant gaps in service provision, particularly regarding substance use treatment and mental health support.

**Conclusions:**

Using a person-centred approach, this article offers a novel way of analysing care contacts among people who subsequently died from opioid overdose. The identification of distinct groups, particularly a large group of people with minimal contact with the community care system, highlights the need for more targeted outreach and support work. Developing targeted interventions in emergency and inpatient care settings may provide an opportunity to reach the group with few care contacts.

## Introduction

The global rise in opioid addiction and opioid-related mortality over the past two decades poses a major public health challenge [1, 2]. In many countries, fatal opioid overdoses are a leading cause of death among people under 50 years of age [3, 4].

Known risk factors for fatal opioid overdose include male gender, increasing age, injection as a route of administration, the use of potent synthetic opioids, concomitant use of other drugs (especially benzodiazepines and other depressants), the resumption of non-medical use after a period of reduced tolerance (e.g. after leaving treatment or prison), a recent non-fatal overdose, and psychiatric or somatic comorbidity [5–10]. Social risk factors include weak social networks, homelessness, and using drugs alone or in unsafe environments [9–11].

However, there is a dearth of research concerning the lives of deceased individuals who used opioids during the time leading up to their deaths — particularly with regard to their interactions with addiction treatment services, healthcare, and social services. Knowledge of contacts with such care agencies is crucial to identifying care gaps and developing interventions and strategies to reach at-risk populations.

In a prior registry study on opioid-related fatalities in Scania (Skåne) County, Southern Sweden, two of the authors investigated contacts with the community care system in a retrospective cohort of individuals who had died of opioid overdose over a four-year period [12]. The study revealed that a vast majority of the deceased had engaged with various care agencies in the year preceding their deaths, including 75% with healthcare services, 69% with social services, and 28% with the prison and probation service. In total, 89% had contacts with at least one of the agencies considered in the study during the final year of their lives.

These findings were consistent with the few existing studies at the time [13–16] and are also in line with research published following the completion of our study [17]. Taken together, these studies indicate that people who die from opioid overdose are well known to the community care system and often have extensive contacts with multiple care agencies.

The studies mentioned above all used variable-centred approaches, i.e. they focused on the different care agencies separately and examined the proportions of the deceased who had been in contact with them. Variable-centred analyses are important because they provide information about the most common points of contact with at-risk populations; this is where the likelihood is highest of effectively reaching at-risk individuals with naloxone distribution and other interventions to prevent overdose deaths.

However, there is a risk that the variable-centred approach may overvalue individuals’ occasional contacts with service providers. For example, a person who has made a single visit to a somatic emergency department may be classified as having a “healthcare contact” in the same way as a person who has had extensive contacts with psychiatric care or addiction treatment services.

By using a person-centred approach, this problem can be mitigated. In such an approach, individuals are classified into different categories based on their scores on several different variables simultaneously [18]. This approach allows for the identification of categories where individuals within a group are more similar to each other, while those in different categories differ more significantly.

In this study we conduct a person-centred re-analysis of the data used in the previous study [12]. The aim is to identify distinct groups among the deceased, based on their contacts with different care agencies and services in their final year of life. Given the categorical nature of our data, we apply latent class analysis (LCA), a statistical method that identifies unobservable (latent) subgroups within a population based on observed categorical variables [19, 20]. Using person-centred mixture modelling, LCA allows for the identification of the optimal number of latent classes and the assignment of individuals to these classes.

LCA has been favoured for the study of patterns of polysubstance use among people who use drugs [21–24]. It has also been used to classify overdose fatalities according to the drugs present in the body at the time of death [25, 26], and to analyse the role of traumatic experiences for overdose fatalities [27]. However, to our knowledge, LCA has never previously been used to study patterns of care contacts among people who use drugs, either deceased or living.

As we will show, the re-analysis of our data presents a significantly different perspective from that found in previous research on deceased individuals’ prior contacts with the addiction and healthcare system.

## Methods

### Setting

The study is a retrospective registry study based on an extensive dataset from a research project on opioid-related fatalities in Scania County, Southern Sweden. There are no current estimates of the prevalence of opioid addiction in Sweden, but opioid-related deaths increased steadily from 2000 to 2017, and Swedish levels are among the highest recorded in Europe [28]. It should however be noted that Sweden has not experienced an American-style opioid crisis, i.e. one triggered by an increase in the supply of prescription opioids. In fact, opioid prescribing has remained relatively stable since the 1990s [29]. Instead, the increase in opioid-related mortality began in the mid-1990s, initially as a result of the introduction of heroin to new locations outside the major cities [30].

### Data collection and sample

The material includes a complete census of fatalities from two periods spanning a total of four years: January 1, 2012, to December 31, 2013, and July 1, 2014, to June 30, 2016. Opioid-related fatalities were identified through a review of records at the National Board of Forensic Medicine in Lund. All forensically examined deaths were manually reviewed, a total of around 4,000 examinations over the four years. Opioids were detected in 503 cases.

The inclusion criteria for this study are: (1) age 18–65 years, (2) a documented history of illicit substance use or presence of injection marks in the autopsy report or police investigation, and/or the detection of an illegal drug (heroin/6-monoacetylmorphine, amphetamine, cocaine, or THC, tetrahydrocannabinol) in the forensic analysis, and (3) cause of death being acute intoxication, and where an opioid (buprenorphine, fentanyl/fentanyl analogs, heroin, methadone, morphine, or oxycodone) was deemed to have been of decisive or essential importance for the death. All three criteria had to be met for cases to be included. In total, 191 deceased individuals were included in the study. The previous study [12] comprised 180 cases and focused on comparisons between deaths resulting from heroin, methadone, buprenorphine, and fentanyl. This study also includes morphine and oxycodone, which were present in too few cases per substance to be included in comparisons in the earlier study. We refer to previous work for detailed information on the data collection process [12, 31].

The study has been subject to ethical review and was approved by the Regional Ethical Review Board in Lund (case no. 2014/547; 2015/369 and 2016/771).

### Data sources and measures

The records from the National Board of Forensic Medicine include demographic data on the deceased and information on causes of death, on the presence and quantity of various substances identified through toxicological analysis, and on certain circumstances related to the death, such as the location and presence of witnesses.

In addition to data from the Swedish National Board of Forensic Medicine, information regarding contacts in the year preceding death was collected from regional healthcare services, municipal social services, and the Swedish Prison and Probation Service. This comprehensive approach sets this study apart from most prior research, which has primarily relied on data from healthcare registers. Collecting social services data was particularly labor-intensive, involving personal contacts and visits to all 33 municipalities in the Scania region (there are no national or regional social services registers in Sweden).

The linkage of data sources was based on the unique personal identification number assigned to every Swedish resident [32].

#### Data sources and predictors for the latent class analyses

The determination of latent classes was based on predictor variables referring to types of contacts with various care agencies. All variables are dichotomous, and refer to contacts in the year preceding death, with the exception of “Previous addiction treatment” (see description below).

Healthcare is regionally organized in Sweden, with a regional council overseeing the healthcare system in Scania County, irrespective of public or private provision. Data on emergency and inpatient contacts with somatic and psychiatric/addiction hospital care are included, as well as opioid agonist treatment (OAT). The review of medical records did not cover primary care or needle exchange programs. The dataset does not include contacts noted in medical records that occurred in connection with death (for example, emergency care admission for an overdose that resulted in death).

The variable “Opioid agonist treatment” refers to ongoing or discontinued OAT. “Inpatient psychiatric or addiction care” and “Inpatient somatic care” refer to any psychiatric/addiction or somatic inpatient healthcare. Likewise, “Emergency care, psychiatric or addiction” and “Emergency care, somatic” refer to any such emergency care. “Non-fatal overdose” was identified from ICD codes indicating intoxication in medical records. “Attempted suicide” was identified through written information in medical records noting an attempted suicide in the year preceding death.

The municipal social services are responsible for providing non-medical addiction treatment and social support for individuals with substance use problems. Services include both outpatient and residential treatment. Treatment is usually voluntarily initiated when an individual seeks assistance from the local social services. However, compulsory care may be imposed in severe cases, where serious medical or social complications are likely and the individual does not agree to voluntary care. The social services also manage the provision of social support, housing services, and financial assistance for those who lack the means to support themselves.

The variable “Social services, addiction unit” refers to any contact with the social services addiction unit (investigation and/or intervention). “Residential treatment” and “Housing services” indicate that the individual received residential addiction treatment or any form of housing intervention from the social services. “Compulsory treatment investigation” means that an investigation had been initiated in accordance with compulsory care legislation. “Previous addiction treatment” indicates experience of non-medical addiction treatment prior to the final year of life.

The Swedish Prison and Probation Service is the national authority responsible for implementing prison sentences and community supervision. Rehabilitation programs for substance addiction are offered during incarceration and periods of supervision. “Probation service” refers to ongoing or completed supervision in the form of probation or upon conditional release from prison, while “Prison sentence served” indicates that the individual served a prison sentence.

#### Measures for comparative analyses between latent classes

The variables used to analyse potential differences between the classes identified in the LCA are described below. All variables are dichotomous with the variable values “yes” and “no”, except for “sex” (male/female), and “age” (continuous).

“Financial assistance” indicates having received financial assistance from the social services in the final year of life. “Stable housing” refers to own housing, sub-tenancy housing, stable accommodation, or housing with support and service for individuals with certain functional disabilities; unstable housing, in contrast, includes homelessness, residence in a treatment facility, shelter, hotel, drug-free communal housing, or an unknown housing situation. “Death in own residence” means that the fatal intoxication occurred in the person’s own residence, as mentioned in “stable housing” above; other typical places where death occurred were in someone else’s home or in public places, hotels, treatment facilities, or homeless shelters. “Witness present” means that someone was present and awake at the place and time of death.

“Needle marks” refers to the presence of injection marks in forensic records. “Benzodiazepines (total)” indicates the presence of any benzodiazepine in forensic analyses. “Z-drugs” refers to the presence of zopiclone; zolpidem was not present in the material. “CNS stimulants” indicates the presence of amphetamine, cocaine, MDMA or methylphenidate. “Alcohol” indicates a blood alcohol concentration above 0.5‰ (to avoid cases where alcohol had been produced in the body after death). “Antidepressants” indicates the presence of prescription antidepressant medications. In addition to the above variables, we have included the opioids that constitute the focus of the study, as well as all other substances with a prevalence > 10% in the population.

Descriptive statistics for all variables included in the study are presented in Table 1.

**Table 1:** Descriptive statistics for study variables

*Contacts with care agencies*	%	*N*
Opioid agonist treatment	22.5	43
Inpatient psychiatric or addiction care	31.4	60
Inpatient somatic care	36.6	70
Emergency care, psychiatric or addiction	31.9	61
Emergency care, somatic	35.1	67
Non-fatal overdose	31.9	61
Attempted suicide	9.9	19
Social services, addiction unit	57.1	109
Residential treatment	20.9	40
Housing services	18.3	35
Compulsory treatment investigation	15.2	29
Previous addiction treatment	51.8	99
Probation service	28.3	54
Prison sentence served	8.4	16
*Variables used in comparative analysis*		
Age (mean, SD)	35.45	10.75
Sex (male)	82.2	157
Financial assistance	50.8	97
Stable housing	70.2	134
Death in own residence	58.6	112
Witness present	40.8	78
Needle marks	46.6	89
Heroin	18.3	35
Morphine	26.2	50
Codeine	22.5	43
Methadone	47.1	90
Buprenorphine	27.2	52
Fentanyl	16.2	31
Oxicodone	5.8	11
Benzodiazepines (total)	72.3	138
Alprazolam	45.0	86
Diazepam	16.8	32
Pregabalin	24.6	47
Z-drugs	23.0	44
CNS stimulants	23.0	44
Alcohol	17.8	34
Cannabis (THC)	19.9	38
Antidepressants	25.7	49

### Statistical analyses

To identify unobservable groups among those who died as a result of fatal opioid overdose, latent class analyses (LCA) were performed (Nylund-Gibson & Choi, 2018; Weller et al., 2020). The determination of latent classes was based on dichotomous variables referring to types of contact with various care agencies during the final year of life. As predictors we included all variables presented in the section “Data sources and predictors for the latent class analyses” above. The R package poLCA (Polytomous Latent Class Analysis) was used, and analyses with different numbers of classes were compared. The maximum Log-Likelihood, Akaike’s Information Criteria, the Bayesian Information Criteria, and entropy R^2^ were used to test the models’ fit. The model with interpretable classes and the lowest BIC value was chosen.

Differences between the identified classes were analyzed using Fisher’s Exact Test for categorical variables and Anova for the continuous variable “age”. The statistical analyses were performed in IBM SPSS Statistics 28 for Windows (IBM Corporation, Armonk, NY, USA) or R Core Team (2022). R: A language and environment for statistical computing. R Foundation for Statistical Computing, Vienna, Austria. A p-value below 0.05 was considered statistically significant.

## Results

### Determination of latent class membership

We compared the fit indices (Maximum Log-Likelihood, Akaike’s Information Criteria, Bayesian Information Criteria, and entropy R^2^) and class sizes for three latent class models. The fit statistics are presented in Table 2. We chose the three-class model, which had easily interpretable classes and the lowest BIC value.

**Table 2:** Comparison of fit statistics across LCA models for 14 binary indicators of contacts with various care agencies

No. of classes	LL	AIC	BIC	Entropy *R*^2^
2	-1322.43	2702.85	2797.17	0.90
**3**	**-1279.47**	**2646.95**	**2790.05**	**0.87**
4	-1250.32	2618.65	2810.53	0.85

LL = Maximum Log-likelihood; AIC = Akaike’s Information Criteria; BIC = Bayesian Information Criteria

**Fig. 1:**
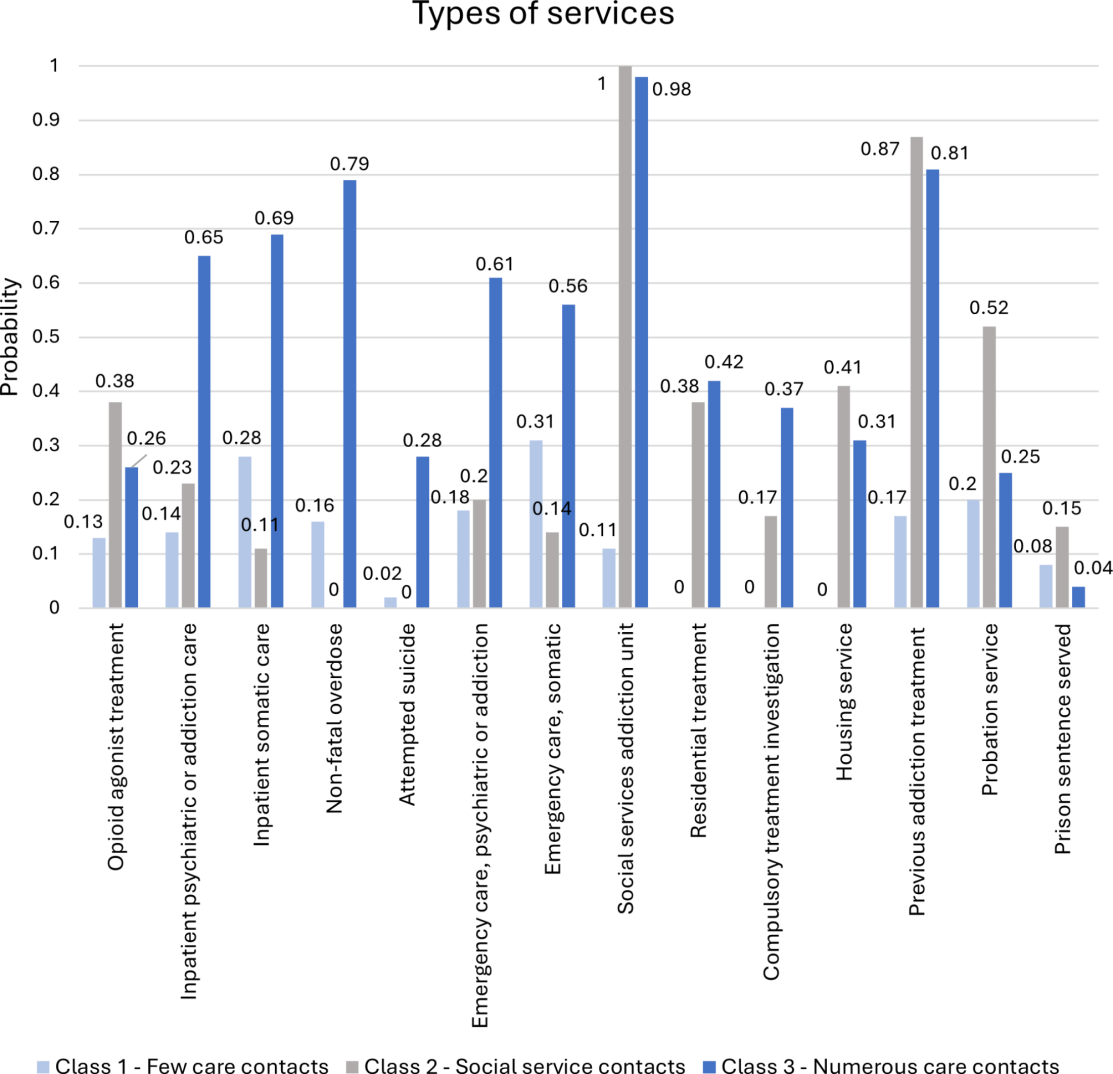
Probabilities of using different types of care and services for each class in the 3-class solution

Figure 1 illustrates the conditional probabilities that the deceased in each class had contact with different types of care and services in their final year of life. Class 1 is the largest, representing 48% of the sample (*n* = 90). This class is characterised by consistently having few care contacts; the contacts they did have were mainly with somatic healthcare, both acute (31%) and inpatient (28%). Recent contact with the social services was virtually non-existent, and the probability of having previously received addiction treatment from the social services was only 17%. Just over one fifth (21%) of the members of this class had no contacts at all with the care agencies included in the study.

Class 2, representing 21% of the sample (*n* = 42), is characterised by high probabilities of social services contact; 100% for some form of addiction treatment through the social services, 38% for residential treatment, and 41% for housing services. The probabilities were also high – 52% and 15% respectively – that the members of this class had been in contact with the probation service or had served a prison sentence in the last year of their lives. In contrast, relatively few in this class had contact with healthcare services, except for a 38% probability of having received opioid agonist treatment.

Class 3, representing 31% of the sample (*n* = 59), is characterised by numerous care contacts. In terms of social services contact, this group does not differ much from Class 2, except for a high probability (37%) of being subject to a compulsory care investigation in the final year of life. The major difference concerns contacts with healthcare services. For members of Class 3, the conditional probabilities were substantially higher for having had contact with psychiatric healthcare services, both acute (61%) and inpatient (65%), and the probability of having been recorded for an attempted suicide was 28%. Class 3 also had extensive contacts with somatic healthcare; the probabilities were 79% for having been treated for a non-fatal overdose, 56% for having received other somatic emergency care, and 69% for having received inpatient somatic care.

### Characteristics associated with different class membership

Table 3 presents comparisons of demographic, substance-related, and other forensic data between the three identified classes.

**Table 3:** Demographic, substance-related, and forensic characteristics across the three identified classes

	Class 1: Few care contacts *n* = 90	Class 2: Social service contacts *n* = 42	Class 3: Many care contacts *n* = 59	*P*-value
Age (mean)	35.64	33.37	37.63	0.107
Sex (male)	81.1%	81.0%	84.7%	0.882
Financial assistance	31.1%	69.0%	67.8%	< 0.001
Stable housing	80.0%	61.9%	61.0%	0.018
Death in own residence	71.1%	45.2%	49.2%	0.004
Witness present	36.7%	47.6%	42.4%	0.484
Needle marks	41.1%	64.3%	42.4%	0.038
Heroin	16.7%	16.7%	22.0%	0.692
Morphine	24.4%	28.6%	27.1%	0.847
Codeine	17.8%	26.2%	27.1%	0.321
Methadone	42.2%	52.4%	50.8%	0.371
Buprenorphine	28.9%	21.4%	28.8%	0.658
Fentanyl	15.6%	21.4%	13.6%	0.565
Oxicodone	6.7%	2.4%	6.8%	0.668
Benzodiazepines (total)	68.9%	66.7%	81.4%	0.165
Alprazolam	56.7%	57.1%	49.2%	0.757
Diazepam	15.6%	11.9%	22.0%	0.411
Pregabalin	15.6%	23.8%	39.0%	0.006
Z-drugs	20.0%	21.4%	28.8%	0.447
CNS stimulants	17.8%	33.3%	23.7%	0.148
Alcohol	20.0%	19.0%	13.6%	0.643
Cannabis (THC)	14.4%	23.8%	25.4%	0.201
Antidepressants	28.9%	19.0%	25.4%	0.485

All tests conducted using Fisher’s Exact Test, with the exception of age, where Anova was used

Gender and age distributions did not differ between classes, but having received financial assistance was more common in Classes 2 and 3 than in Class (1) Stable housing at the time of death and death in one’s own residence were more common in Class 1 than in Classes 2 and 3, where the majority of deaths occurred elsewhere. Regarding the substances identified in blood samples at forensic examination, there were generally few differences between the classes. Benzodiazepines and other sedatives were very common in all classes, but the deceased in Class 3 had a higher prevalence of pregabalin than Classes 1 and (2) Class 2 was more likely than Classes 1 and 3 to have fresh needle marks.

The between-class comparisons are exploratory in nature and we do not aim to provide an explanatory analysis of different class memberships. We have therefore not used any method to correct for multiple comparisons.

## Discussion

Previous variable-centred research has indicated that people who die from opioid overdoses are largely known to the community care system and often have extensive contacts with care agencies during the recent period prior to death [12, 13, 15–17]. This study, based on a person-centred approach and using LCA, challenges these findings. The analysis identified three distinct groups among the deceased, based on their care contacts in the year preceding their deaths.

Class 1 (Few care contacts) comprises people who use opioids and who appear to be largely “flying under the radar”, a group that was not visible in our previous, variable-centred analysis [12]. These are people who have little or no contact with care agencies and are therefore likely to be harder to reach than other groups in terms of interventions to prevent overdoses and other drug-related deaths. When individuals in this group do seek care, it is primarily somatic healthcare. The comparative analysis showed that members of this class were more likely to have died in their own homes and that they displayed relatively stable social and economic circumstances, as evidenced by a high level of stable housing and less reliance on financial assistance.

Research on people who use opioids in socially integrated settings and on those who do not seek help from care agencies despite high-risk opioid consumption is scarce. Existing studies indicate that people who use opioids in socially integrated settings often lead relatively structured lives, with employment or education, and often have stable housing [33–37], which corresponds with our Class 1 characteristics. For most health conditions, one would expect that more stable social and economic circumstances would increase the likelihood of individuals accessing care. However, there may be a variety of reasons why people who use opioids do not typically seek or access care. They may consider their drug use to be under control and as not requiring treatment, or they may fear stigmatization and legal repercussions. Additionally, some may not be aware of the availability of treatment options or believe that they can manage their drug use without professional intervention [33–37].

Classes 2 and 3 appear to be more “clinical” groups of people who use illicit opioids, i.e. people who have frequent contact with addiction treatment services. Class 2 (Social service contacts) were well known to the social services and had a high level of criminal activity, as indicated by their high number of contacts with prison and probation services. In contrast, members of this class had fewer contacts with healthcare services, apart from the fact that a relatively high proportion of this group had recent experiences of OAT.

Class 3 (Numerous care contacts) is a very care-intensive group with extensive polysubstance use and high levels of psychiatric comorbidities. This group was well known to the community care system, including both psychiatric and somatic health services and the social services. Many in this group had experienced non-fatal overdoses or attempted suicide in the year before their death. Relatively many had also been subject to a compulsory care investigation, despite having contact with voluntary care. This suggests that many members of this group were living in difficult or chaotic conditions before they died.

Classes 2 and 3 are likely to mirror the cohorts described in clinical addiction treatment research. Clinical studies show that patients often cycle in and out of treatment, with periods of abstinence and frequent relapses [38, 39]. Many live under difficult social conditions involving unstable housing or homelessness [38, 40]. To buy drugs, they often use a variety of income sources, including crime and financial assistance [41–43].

Psychiatric comorbidities, such as anxiety, depression, traumatic experiences, and personality disorders, are highly prevalent in clinical populations of opioid-dependent individuals [44–49]. Our findings are consistent with this, especially with regard to Class 3, in which psychiatric comorbidities appear to be the defining trait. Psychiatric comorbidities are often associated with various adverse treatment outcomes [46, 49–52], including fatal overdoses [51, 53]. However, the risk of death is generally high in clinical populations of people who use opioids [51, 54–56].

In the between-class comparative analysis, there were very few differences between the classes in terms of the drugs found in the body at the time of death. This is interesting because it suggests that Class 1, which had a more stable social situation than Classes 2 and 3, had equally extensive and risky drug use. There were only two significant differences between the classes. The fact that a larger proportion of those in Class 2 had fresh needle marks is difficult to interpret and may be a coincidence. However, the fact that a larger proportion of those in Class 3 had pregabalin in their blood is probably due to the intensive healthcare contacts in this class. During the data collection period, pregabalin was frequently used as an anxiolytic in patients with substance use disorders. Pregabalin was classified as a narcotic drug in Sweden in 2018, and prescriptions have since decreased.

In a previous paper, using data from the Swedish Prescribed Drug Register, we showed that pregabalin and z-drugs were the only commonly prescribed drugs for which a small majority of the deceased had had a prescription in the last six months of their lives. All other prescription drugs identified in the toxicological analysis had predominantly been used illicitly [57].

In this context, it can be noted that a substantial number of the members of all three classes of deceased individuals had methadone or buprenorphine, the drugs used in opioid agonist treatment, in their bodies. Indeed, in all three classes, methadone was the opioid that was most frequently detected. However, as shown in Table 1, only 22.5% of the deceased had received OAT in their final year of life. Diversion and the illicit use of OAT drugs has been recognized as a problem in Sweden [58, 59]. Previous studies have shown that 75–80% of those who die with these substances in their body have no ongoing OAT [60, 61], which is consistent with our findings. OAT drugs are often used for self-medication purposes by people with opioid dependence, but such use is associated with significant risks, including the risk of death [62, 63].

Given the well-documented effectiveness of opioid agonist treatment in reducing opioid-related mortality [64, 65], ensuring that this treatment modality is readily accessible, and preventing discontinuation or involuntary discharge, is paramount in reducing the risk of overdose deaths across all identified classes. Apart from this, and in the light of our findings, targeted interventions may be crucial to effectively preventing deaths among the different classes of people who use opioids. Class 2 may benefit from housing interventions, since many lack stable housing. This is particularly true for interventions such as Housing First, which are considered effective in helping people with substance use or mental health problems but are very difficult to access in Sweden [66]. To prevent deaths in Class 3, both OAT and housing interventions are important, but above all the members of this class seem to need more effective care for their psychiatric comorbidities. This could be achieved by implementing coordinated care models that integrate treatment for substance use disorders with mental health services.

Class 1 presents a challenge as a result of their minimal interaction with the community care system. When individuals in this class appear in the healthcare system, it is often because of somatic morbidity, in emergency departments but also in inpatient care. Developing targeted screening methods in emergency and inpatient care settings may therefore constitute an important means of reaching this group. Screening methods could aim to identify individuals at risk in order to provide information on overdose prevention, administer take-home naloxone, and offer referral to OAT or other addiction treatment services [17]. The class members’ contacts with the social services were mainly focused on financial assistance; here too there may be some potential for identifying individuals at risk for opioid overdose.

This study has certain limitations that should be acknowledged. To begin with, since this is a re-analysis of previously published data, the data are rather old. However, there is no evidence that the data are outdated; as already mentioned Sweden does not have an ongoing opioid crisis like the US, and the annual drug-related mortality rate has remained relatively unchanged since the period during which our data were collected. The Swedish care system for people with substance use problems has also not changed significantly in recent years, except for the introduction of naloxone programs in most regions. Another notable limitation is the relatively small sample size. Although there is no strict threshold for the number of cases in which LCA can be used, there are risks associated with a small sample, particularly the risk of not detecting small classes that may be hidden in the data [19]. Although the classes that emerged from the analysis were meaningful and easy to interpret, it would be interesting to perform similar analyses on larger data sets in the future. The absence of data on primary care contacts is also a limitation. Previous research has highlighted that frequent contact with primary care providers is common among individuals who later succumb to overdose [13, 15, 17]. However, since the responsibility for the treatment of substance use disorders in Sweden is shared between the social services and specialised psychiatry, we do not consider the lack of primary care data to be a major shortcoming of our study. Additionally, our study lacks information on any contacts with care agencies that may have occurred prior to the final year of the subjects’ lives. This is particularly relevant for individuals in Class 1, as it is plausible that they may have had such contacts.

## Conclusions

This study presents a novel perspective on the care contacts of individuals who died from opioid overdoses, challenging previous findings in this area. The discovery of distinct groups among the deceased – particularly a significant group with minimal interactions with the community care system – underscores the need for more targeted outreach and support work. The findings emphasize the importance of designing interventions that are specifically tailored to meet the diverse needs of different at-risk populations.

## Data Availability

The SPSS dataset used in the current study is not publicly available due to restrictions made by the Regional Ethical Review Board in Lund, Sweden, but is available from the corresponding author on reasonable request.

## Abbreviations

LCA: Latent class analysis
OAT: Opioid agonist treatment
